# Are we over-dependent on pharmacotherapy?

**DOI:** 10.4103/0019-5545.39750

**Published:** 2008

**Authors:** Roy Abraham Kallivayalil

**Affiliations:** Department of Psychiatry, Government Medical College, Thrissur - 680 596, Kerala, India

“It has become common practice for pharmaceutical companies to have all of the key articles linked to their drugs ghostwritten. Up to 50% of articles on therapeutics appearing even in the most prestigious Journals such as the Lancet and the New England Journal of Medicine may now be ghostwritten. A general problem with this is that such articles will not deviate from the company line. A more specific problem is the evidence indicating that company articles on therapeutics often fail to refer to the data on serious hazards of treatment such as suicidal acts or indeed evidence that not only raw data has been suppressed, but it has in some instances been significantly changed.”

- David Healy (2005)[1]

The above criticism certainly urges us to be cautious in our clinical practice. It is believed, an increasing proportion of such ghostwritten, so-called scientific literature in therapeutics is ornamental rather than substantive. Hence, is it correct if we conduct clinical practice based only on such information? Are not the perceptions of our patients important? Are they not different in India when compared to the West? It has to be stated with emphasis, too much of academic certainty is enemy of science and progress.

Our clinical practice should consider patients' experiences and try not to invalidate them. Many times, our treatment might make the patient worse than before. In such instances, increasing the medication or adding a new drug are the options we generally consider. This is often done without carefully listening to the patient, as many of us are extremely busy. We are tempted to believe, drugs are the remedy for all human sorrows and difficulties. We know, a drug which is curative for one may be dangerous for another. Hence, over-reliance on medication can be extremely harmful.

Intuitively, we expect uniform response for a particular drug in our patients. But we fail to remember, people have different proportions of receptors for each drug to act upon and no such response is possible. We often add new drugs to the existing regimen when there is lack of adequate response. We are tempted to blame our patients for poor drug compliance, whereas the problem may in fact be due to overprescribing of antipsychotics! Are we not falling prey, at least sometimes to the promotion strategies of drug companies? Pharma companies can escape liability since they might have provided warnings and list of side-effects. In the past, companies have termed suicidal acts as emotional instability and withdrawal symptoms as symptoms due to stopping the prescribed medication! In the end, the treating psychiatrist will have to face the blame. This is an important factor to be considered when prescribing in our country, where more and more doctors are facing consumer litigation.

Suicides following antipsychotic- or antidepressant-induced akathisia have been reported. Clinicians know, SSRIs can induce akathisia, but companies deny such a side-effect. The case of fluoxetine is a classical example. We know, fluoxetine can lead to akathisia and agitation. In fact, benzodiazepines were recommended as co-prescription during clinical trials of the drug. This akathisia may lead to violence and suicide. But the company's database has no mention of akathisia! Many authors believe, fluoxetine may not only induce suicidal ideation early in treatment and suicidality in some, but also diminish emotional reactivity. The company literature places sexual dysfunction at only 5%, but clinical experience shows much higher incidence.

Tardive dyskinesia is a long-term and sometimes unavoidable complication of long-term antipsychotic use. But how will we explain if the antipsychotic had been prescribed for anxiety or as a hypnotic? We have to accept that no drug is safe. In practice, we will have to consider the possible benefits and the potential risks. If the risks are likely to be more, we have to exercise extreme caution.

Another happening is that company representatives are penetrating into patient groups. This has already happened in the West and is probably happening in India. The question asked by such patient groups is, why should patients be denied the latest and most effective treatment just because the drugs are costly? One can easily see the powerful marketing strategies for newer drugs. But new evidence is now emerging, patients might do equally well, even marginally better, with older antipsychotics as well.

We are all aware about the CATIE trial, wherein Lieberman *et al.*,[2] investigated the effectiveness of antipsychotic drugs in patients with chronic schizophrenia and found, the conventional antipsychotic perphenazine was as effective as second generation (atypical) antipsychotics olanzapine, quetiapine, risperidone and ziprasidone. Only Olanzapine was more effective in terms of rate of discontinuation. This will bring the question of cost-effectiveness back to focus especially in the developing world.

Jeffrey Lieberman wrote, “The claims of superiority for the [newer drugs] were greatly exaggerated. This may have been encouraged by an overly expectant community of clinicians and patients eager to believe in the power of new medications. At the same time, the aggressive marketing of these drugs may have contributed to this enhanced perception of their effectiveness in the absence of empirical information.”

In the UK, the House of Commons Health Committee in its report ‘The Influence of Pharmaceutical Industry’ (2005) said, “The aggressive promotion of medicines shortly after launch, the sheer volume of information that is received in its many forms by prescribers” and the “promotional hospitality masquerading as education”, in the absence of effective countervailing forces - all contribute to the inappropriate prescription of medicines (Paragraph 232).

Another study, funded by the British government, is the first to compare treatment results from a broad range of older antipsychotic drugs against results from newer ones. The study was requested by Britain's National Health Service to determine whether the newer drugs - which can cost 10 times as much as the older ones - are worth the difference in price. The study, published in the Archives of General Psychiatry,[3] is likely to add to a growing debate about prescribing patterns of antipsychotic drugs. In the study, 227 schizophrenia patients were randomly assigned to two groups - one group received a newer Second Generation Antipsychotic (other than clozapine), the other an older First Generation Antipsychotic drug. The patients were evaluated for more than a year. There was no difference in Quality of Life (QOL), although newer antipsychotics costed 10 times more. Influential newspaper Washington Post wrote, “The results are causing consternation. The researchers who conducted the trial were so certain they would find exactly the opposite that they went back to make sure the research data had not been recorded backward”.

And in 2006, the US FDA approved the sale of placebo prescriptions which were given after more than four decades of testing in tandem with other drugs. “For years, scientists have been aware of the effectiveness of placebo in treating a surprisingly wide range of conditions,” said Dr. Jonathan Bergen of the FDA's Center for Drug Evaluation and Research. “It was time to provide doctors with this often highly effective option.”

In summary, we may state that drug treatment alone - despite the vast advances in psycho-pharmacology - still remains unsatisfactory. Psychiatry is a bio-psycho-social speciality. We have to consider the biological, psychological and sociocultural aspects in diagnosis and treatment. APA guidelines on depression states, “Psychotherapeutic management is an essential component of every medication based treatment plan”. In the US - which we often try to emulate - most psychiatric patients receive both medication and psychotherapy. In managed care arrangements, often paid by insurance companies, the services may be delivered by two professionals. At other times, a single psychiatrist provides integrated treatment in a systematic way.

What can be the answer in the situation in India? Combining supportive psychotherapy with pharmacotherapy might well be the answer. Unfortunately, in our country, psychotherapy has been neglected for long. Even in the book, *Mental Health - An Indian Perspective*,[4] which deals with the psychiatric services in post-independent India, psychotherapy has largely been left out. There are only few centres in the country which give adequate training and emphasis in psychotherapy even during post-graduate training. This might lead to some of our young psychiatrist-colleagues woefully lacking in psychotherapeutic skills.

We know that a significant number of patients on placebo in clinical trials show improvement probably due to psychotherapeutic factors. A proper evaluation of the symptoms and history can make the patient aware of the relationship between external events and his illness, and help him gain insight. A caring psychiatrist, who is willing to listen and provide help, is a positive emotional experience for all patients. Tenets of psychotherapy like transference, resistance, counter-transference etc are integral to pharmacotherapy as well. Patient non-compliance may be due to one of the above factors. Good listening skills, rapport and therapeutic alliance are essential for treatment success.

Increasing cost of medical and psychiatric care in developing countries like India is also a matter of concern. Prescribing too many drugs is not good practice. And giving costly drugs for economically disadvantaged sections when cheaper alternatives are available should be discouraged. It may be worthwhile to quote Amartya Sen, “India offers high quality medical facilities to the Indian rich and to rich foreigners, but basic health services in India are quite bad, as we know from elaborate criticisms of these services in the Indian media”.

This is not against prescribing adequately for our patients. But let us refrain from indiscriminate prescriptions and over-medicalization for all emotional problems. We must know, psychotherapy should be an essential skill for all psychiatrists, especially when drugs do not seem to provide all the answers. Dysfunctional brain in a distressed mind is a model which can be helpful.
